# Bromodomain Protein BRD4-Mediated Mutant p53 Transcription Promotes TNBC Progression

**DOI:** 10.3390/ijms232315163

**Published:** 2022-12-02

**Authors:** Julie Xia Zhou, Ewud Agborbesong, Linda Xiaoyan Li, Xiaogang Li

**Affiliations:** 1 Department of Internal Medicine, Mayo Clinic, Rochester, MN 55905, USA; 2 Department of Biochemistry and Molecular Biology, Mayo Clinic, Rochester, MN 55905, USA

**Keywords:** BRD4, p53, triple-negative breast cancer

## Abstract

*TP53* is the most common mutated gene in human cancer. Mutant p53 protein loses its tumor-suppressor properties and gains oncogenic activity. Mutant p53 is a therapeutic target in a broad range of cancer types. However, how mutant p53 is epigenetically regulated during tumor progression remains elusive. In this study, we found that the upregulation of mutant p53 is mediated by bromodomain protein BRD4 in triple-negative breast cancer (TNBC) cells. Inhibition of BRD4 with its inhibitor JQ1 or knockdown of BRD4 suppressed the transcription of mutant p53, which led to the re-expression of p21, the inhibition of S-phase entry, and colony formation in TNBC cells. BRD4 also positively regulated the transcription of wild-type p53, whereas JQ1 treatment and knockdown of BRD4 decreased the expression of p21 in MCF-7 cells. Knockdown of BRD4 resulted in attenuation of TNBC tumor growth in vivo. Taken together, our results uncover a novel regulatory mechanism of mutant p53 via BRD4, and suggest that the bromodomain inhibitor suppresses tumorigenesis through targeting mutant p53 in TNBC.

## 1. Introduction

The tumor-suppressor gene *TP53* is the most intensively studied gene and is mutated in approximately 50% of all human malignancies and in more than 80% of cases of TNBC [1]. p53 suppresses cancer formation and progression by the processes involving cell cycle arrest, apoptosis, senescence, and DNA repair. Recent evidence indicates that p53 may also protect cells from malignant transformation by regulation of metabolism, reactive oxygen species levels, non-coding RNAs expression, autophagy, or ferroptosis [2]. The wild-type p53 acts as a homotetrameric transcriptional factor to bind with specific DNA sequences and regulates gene expression. Mutations of *TP53* are predominantly missense, and approximately 95% are located in the DNA-binding domain [3]. The mutant p53 proteins with missense mutations in the DNA-binding domain are unable to bind to the p53 specific DNA promoter sites, resulting in loss of tumor suppressor properties and gain of an oncogenic activity [3]. Wild-type p53 is a labile protein with a short half-life, as it is tightly regulated by post-translational modification coupled with proteasomal degradation. However, mutated p53 protein is hyperstable and accumulates in cancer cells [4].

The association of mutant p53 with specific transcriptional factors or chromatin-regulating proteins is one of the mechanisms by which mutant p53 gains oncogenic function. Mutant p53 binds to the cognate DNA-binding sites of those transcription factors and activates gene expression of their downstream pathways, contributing to tumorigenesis and metastasis [4]. In addition, mutant p53 has been suggested to function as a dominant negative inhibitor over any remaining wild-type p53 [4]. Therefore, mutant p53 has emerged as a promising therapeutic target in cancer treatment. A variety of strategies, including promotion of mutant p53 degradation through the proteasome and autophagy pathways, restoration of wild-type p53 activity, interfering with the interaction between mutant p53 and other proteins, and interference in signaling pathways downstream of mutant p53, have been explored [4].

Epigenetic mechanisms have been directly or indirectly involved in the regulation of mutant p53 in cancer cells. It has been reported that the transcription factor homeobox A5 (HOXA5) and p53 bind to the consensus HOX-binding sites and p53-responsive element in the p53 promoter within the region upstream of the transcription initiation site, respectively, to activate p53 expression [5,6]. However, the expression of HOXA5 is regulated by histone deacetylase 8 (HDAC8), which has been suggested to indirectly increase p53 transcription via increasing the binding of HoxA5 on the promoter of mutant p53, thereby promoting tumor cell proliferation [7]. Activation of SIRT1 with YK-3-237 deacetylated mutant p53, resulting in a decrease in the mutant p53 level and inhibition of TNBC progression [8]. In addition, knockdown of enhancer of zeste homologue 2 (EZH2), the catalytic subunit of the PRC2 complex, downregulated the expression of mutant p53 and inhibited the growth of ovarian cancer [9].

Bromodomain-containing protein 4 (BRD4) is a member of the bromodomain and extraterminal domain (BET) family proteins, which recognizes and binds to acetylated-lysine residues on histone tails via bromodomains, and interacts and recruits coactivators and corepressor complexes to the chromatin for regulating gene transcription via its C-terminal domain (CTD) [10]. BRD4 has been reported to regulate the expression of important cell growth and survival genes, such as *MYC*, *BCL2*, *CCND1*, and *FOSL1* [11,12,13,14,15,16]. JQ1, a BET protein small molecular inhibitor, displaces the BET protein from the chromatin and represses gene expression in cancer cells [14]. However, whether BRD4 regulates the expression of mutant p53 in triple-negative breast cancer (TNBC) cells is unknown. In this study, we tested the hypothesis that BRD4 promoted the tumorigenesis through modulating the transcription of mutant p53 in TNBC in both in vitro and in vivo models. 

## 2. Results

### 2.1. BET Bromodomain Inhibitor JQ1 Decreases the Level of Mutant p53 Protein in TNBC Cells 

**Figure 1:**
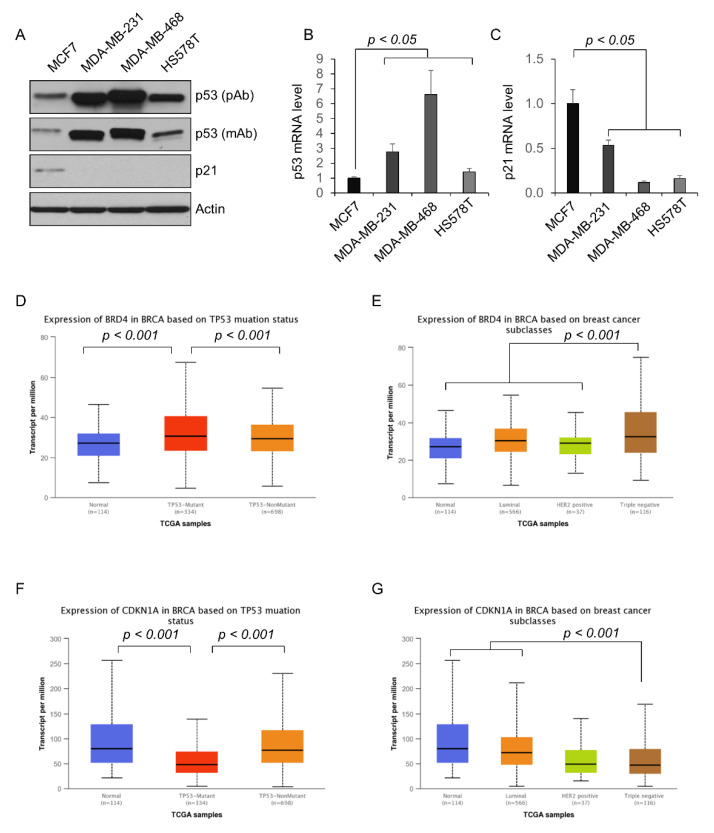
The expression of p53, p21, and BRD4 in breast cancer cells and human breast cancer samples from the TCGA datasets. (**A**) Western blot analysis of the expression of p53 with either a polyclonal antibody (pAb) or a monoclonal antibody (mAb) and p21 protein in MCF-7, MDA-MB-231, MDA-MB-468, and HS578T cells. (**B**,**C**) qRT-PCR analysis of the expression of p53 (**B**) and p21 (**C**) mRNA in MCF-7, MDA-MB-231, MDA-MB-468, and HS578T cells. All data are presented as the mean ± SEM; n = 3, *p* < 0.05 by Student’s *t*-test. (**D**,**E**) The expression of BRD4 in breast cancer samples based on *TP53* mutant status (**D**) or breast cancer subclasses (**E**) from the TCGA dataset analyzed by UALCAN. (**F**,**G**) The expression of p21 (CDKN1A) in breast cancer samples based on the *TP53* mutant status (**F**) or breast cancer subclasses (**G**) from the TCGA dataset analyzed by UALCAN. All data (**D**–**G**) are presented as interquartile ranges (IQR). The n in each group as indicated, and *p* < 0.001 by Welch’s *t*-test.

The estrogen receptor positive MCF-7 cells contain functional p53 (wild-type p53) [17], whereas TNBC cells have a mutated type of p53, including MDA-MB-231 cells with p53-R280K, MDA-MB-468 cells with p53-R273H, and H578T cells with p53-V157F [17]. Consistent with previous reports, the expression of mutant p53 proteins was highly elevated in TNBC cells compared with the level of wild-type p53 in MCF-7 cells as examined by Western blot (Figure 1A). In addition, the levels of mutant p53 mRNA were also increased in TNBC cells compared with the level of wild-type p53 in MCF-7 cells, as examined by qualitative reverse transcription PCR (qRT-PCR) analysis (*p* < 0.05, n = 3) (Figure 1B). MCF-7 cells with wild-type p53 readily expressed a detected level of p21, whereas the expression of p21 protein in p53 mutant TNBC cells was negligible (Figure 1A). The expression of p21 mRNA was also decreased in p53 mutant cells, as detected by qRT-PCR (*p* < 0.05, n = 3) (Figure 1C). We also compared the expression of BRD4 and p21 as well as the association of the expression of BRD4 with mutant p53 in breast cancer samples from the TCGA dataset of the University of Alabama at Birmingham Cancer data analysis Portal (UALCAN) [18,19]. The expression of BRD4 was increased in breast cancer samples compared with normal controls (*p* < 0.001) (Figure 1D). The breast cancer samples with mutant p53 have a higher BRD4 expression compared with those samples with non-mutant p53 (Figure 1D). Additionally, the expression of BRD4 in TNBC is highest among all three subclasses (luminal, HER2 positive, and triple-negative) of breast cancer (*p* < 0.001) (Figure 1E). The expression of p21, also known as CDKN1A, was lower in breast cancer samples with mutant p53 compared with that in normal controls or breast cancer samples with non-mutant p53 (*p* < 0.001) (Figure 1F). The expression of p21 in TNBC is lowest among all three subclasses (luminal, HER2 positive, and triple-negative) of breast cancer (*p* < 0.001) (Figure 1G). 

**Figure 2:**
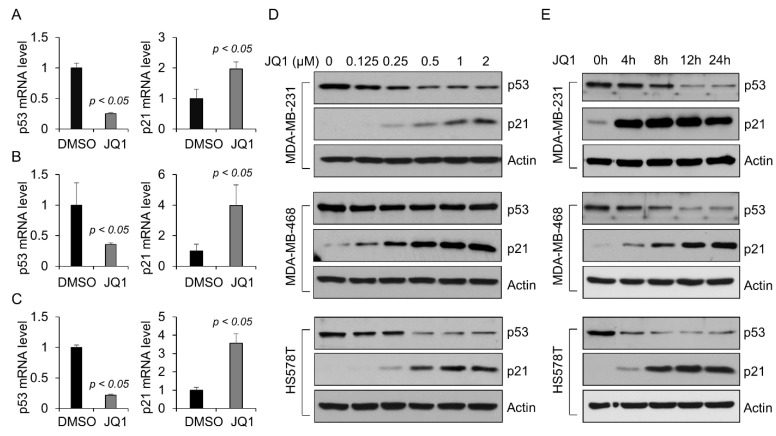
BET bromodomain inhibitor JQ1 decreases mutant p53 protein and increased p21 in TNBC cells. (**A**–**C**) qRT-PCR analysis of the expression of p53 and p21 mRNA in MDA-MB-231 (**A**), MDA-MB-468 (**B**), and HS578T (**C**) cells treated with 1 μM JQ1 for 24 h. All data are presented as the mean ± SEM; n = 3, *p* < 0.05 by Student’s *t*-test. (**D**) Western blot analysis of the expression of mutant p53 and p21 protein in MDA-MB-231, MDA-MB-468, and HS578T cells treated with the indicated concentration of JQ1 for 72 h. (**E**) Western blot analysis of the expression of mutant p53 and p21 protein in MDA-MB-231, MDA-MB-468, and HS578T cells treated with 1 μM JQ1 for 4 to 24 h.

Recently, the BET bromodomain inhibitors (BETi) have been evaluated and shown efficacy in several models of cancer [20]. JQ1 has been proven to be a first-in-class BETi, which can downregulate the transcription of multiple genes through targeting BRD4, such as *MYC*, *IL7R*, and *E2F1* in different cancer cells [21]. To evaluate the effect of JQ1 on the transcription of mutant p53, the MDA-MB-231, MDA-MB-468, and HS578T cells were treated with JQ1. We found that JQ1 treatment decreased the mRNA (*p* < 0.05, n = 3) (Figure 2A–C) and protein (Figure 2D,E) levels of mutant p53 in a dose-dependent and time-dependent manner in these cells, suggesting that the expression of mutant p53 is inhibited at the transcription level by BETi. Knockdown of mutant p53 has been reported to induce the expression of p21 mRNA in T47D cells and nasopharyngeal carcinoma CNE-2 cells [22]. We found that treatment with JQ1 strikingly increased the levels of p21 mRNA and protein in MDA-MB-231, MDA-MB-468, and HS578T cells (Figure 2A–E), which supported that JQ1 treatment not only decreased the expression of mutant p53 but also reactivated the expression of p21 in these cells.

### 2.2. BRD4 Regulates the Transcription of Mutant p53 in Triple-Negative Breast Cancer Cells 

**Figure 3:**
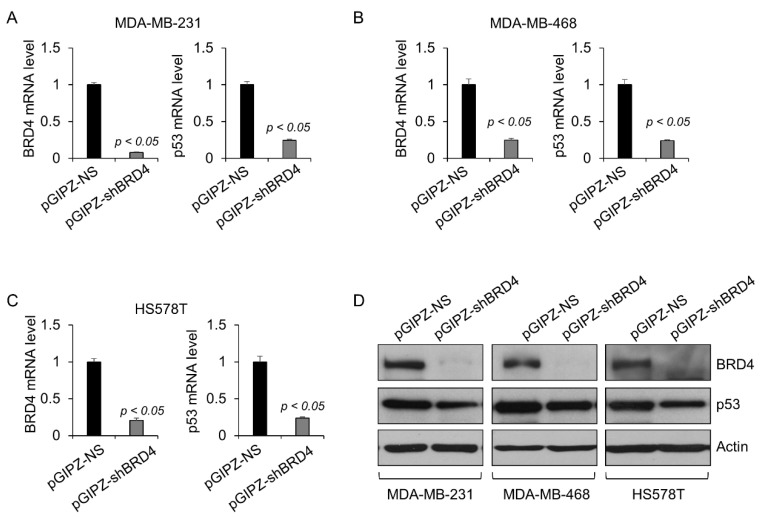
Knockdown of BRD4 decreases the level of mutant p53 transcript and protein in TNBC cells. Western blot and qRT-PCR analysis of the expression of mutant p53 mRNA (**A**–**C**) and protein (**D**) in MDA-MB-231, MDA-MB-468, and HS578T cells transduced with lentivirus expressing BRD4 shRNA (pGIPZ-shBRD4) or control shRNA (pGIPZ-NS). All data (**A**–**C**) are presented as the mean ± SEM; n = 3, *p* < 0.05 by Student’s *t*-test.

To investigate whether the downregulation of mutant p53 upon JQ1 treatment is through targeting BRD4, we knocked down BRD4 with lentivirus-mediated shRNA in MDA-MB-231, MDA-MB-468, and HS578T cells. The knockdown efficiency of BRD4 in these cells was confirmed by qRT-PCR (*p* < 0.05, n = 3) and Western blot (Figure 3A–D). We found that knockdown of BRD4 significantly decreased the expression of mutant p53 mRNA and protein in these cells (Figure 3A–D). These results indicated that BRD4 functioned as a key regulator of mutant p53 transcription in TNBC cells.

**Figure 4:**
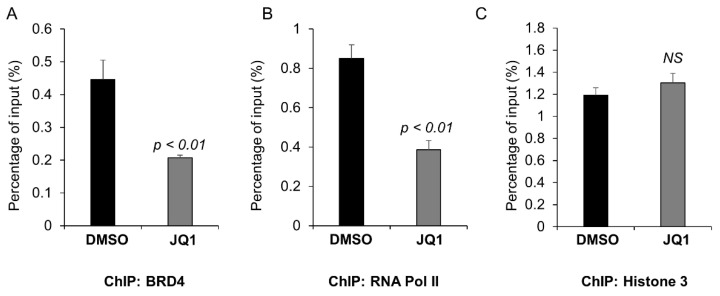
BRD4 regulates p53 transcription through binding with its promoter. A CHIP assay was performed with anti-BRD4 antibody (**A**), anti-RNA polymerase II antibody (**B**), and anti-histone 3 antibody (**C**) in MDA-MB-231 cells treated with DMSO or JQ1 (1 μM) for 16 h. The precipitated chromatin DNA was analyzed by quantitative PCR with primers that were amplified from -200 to +11 bp upstream of the p53 ATG start codon. The percentage of enrichment relative to the input control is shown. All data are presented as the mean ± SEM; n = 3, *p* < 0.01 by Student’s *t*-test. NS, not significant.

To analyze the binding of BRD4 on the mutant p53 promoter, we performed a chromatin immunoprecipitation (ChIP) qPCR assay in MDA-MB-231 cells with and without JQ1 treatment. The ChIP-qPCR results showed that BRD4 were enriched near the transcription start site of the p53 promoter (Figure 4A) and JQ1 (1 μM) treatment decreased the association of BRD4 and RNA polymerase II on the p53 promoter (*p* < 0.05, n = 3) (Figure 4A,B). Anti-histone 3 antibody was used as a negative control, which was not affected by inhibition of BRD4 (Figure 4C). These results suggested that BRD4 directly bound with the promoter of p53 to regulate its transcription in TNBC cells.

### 2.3. BRD4 Regulates the Transcription of Wild-Type p53 in MCF-7 Cells 

**Figure 5:**
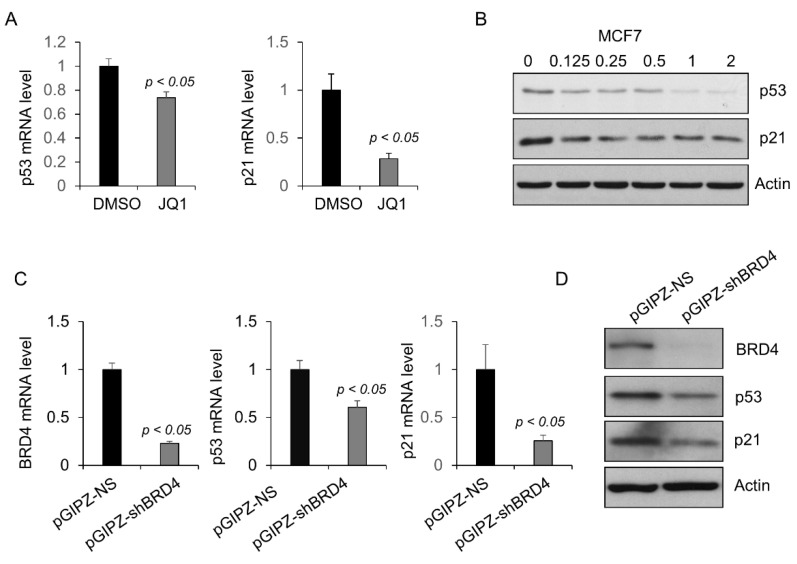
Inhibition or knockdown of BRD4 decreases the level of wild-type p53 and p21 in MCF-7 cells. (**A**) qRT-PCR analysis of the expression of p53 and p21 mRNA in MCF-7 cells treated with 1 μM JQ1 for 24 h. (**B**) Western blot analysis of the expression of wild-type p53 and p21 protein in MCF-7 cells treated with the indicated concentration of JQ1 for 72 h. (**C**) qRT-PCR analysis of the expression of BRD4, p53, and p21 mRNA in MCF-7 cells transduced with lentivirus expressing BRD4 shRNA (pGIPZ-shBRD4) or control shRNA (pGIPZ-NS). (**D**) Western blot analysis of the expression of p53 and p21 protein in MCF-7 cells transduced with lentivirus expressing BRD4 shRNA (pGIPZ-shBRD4) or control shRNA (pGIPZ-NS). All data (**A**,**C**) are presented as the mean ± SEM; n = 3, *p* < 0.05 by Student’s *t*-test.

The promoter sequence of wild-type p53 in MCF-7 cells is the same as that of mutant p53 in TNBC cells, whereas mutations of p53 are only located in the coding region. Consistent with that in the TNBC cells, treatment with JQ1 also decreased the expression of wild-type p53 mRNA (*p* < 0.05, n = 3) (Figure 5A) and protein (Figure 5B) in MCF-7 cells. Knockdown of BRD4 with shRNA also decreased the expression of wild-type p53 mRNA (*p* < 0.05, n = 3) (Figure 5C) and protein (Figure 5D). Interestingly, and contrastingly, inhibition of BRD4 with JQ1 or knockdown of BRD4 with shRNA increased the p21 mRNA and protein in TNBC cells carrying mutant p53 (Figure 2), whereas the same treatments decreased the expression of p21 mRNA and protein in MCF-7 cells (Figure 5A–D). These results further support that mutant p53 has the opposite role as wild-type p53 in regulating gene transcription in breast cancer cells.

**Figure 6:**
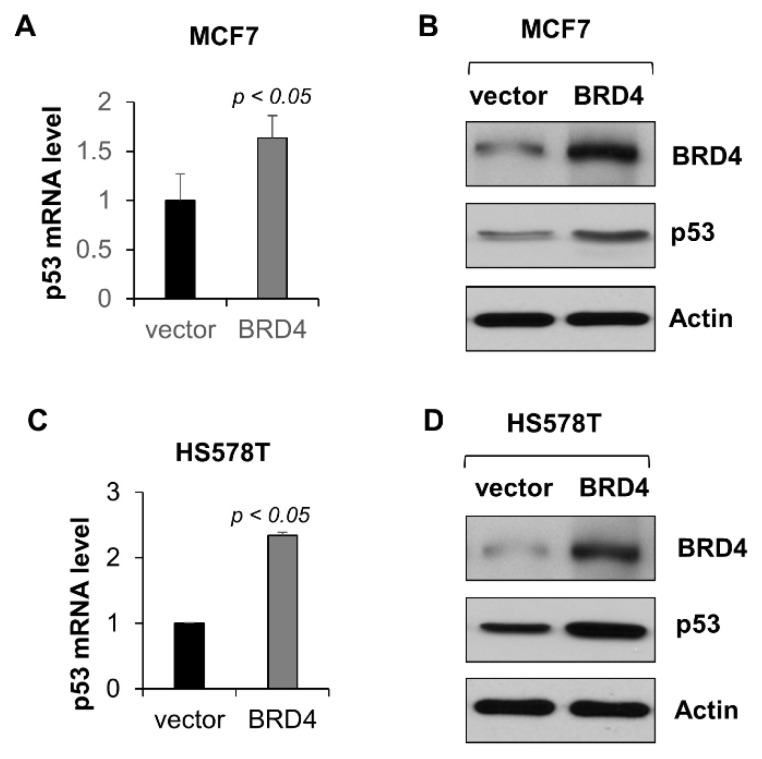
Ectopic expression of BRD4 increases p53 expression. (**A**,**B**) qRT-PCR and Western blot analysis of the expression of p53 mRNA (**A**) and protein (**B**) in MCF-7 cells transfected with pCMV-Flag-BRD4 or control vector for 24 h. (**C**,**D**) qRT-PCR and Western blot analysis of the expression of p53 mRNA (**C**) and protein (**D**) in HS578T cells transfected with pCMV-Flag-BRD4 or control vector for 24 h. All data (**A**,**C**) are presented as the mean ± SEM; n = 3, *p* < 0.05 by Student’s *t*-test.

To further support that BRD4 regulates the expression of wild-type p53, we overexpressed BRD4, and analyzed the expression of wild-type p53 in MCF-7 cells. We found that overexpression of BRD4 increased p53 mRNA (*p* < 0.05, n = 3) (Figure 6A) and protein (Figure 6B) in MCF-7 cells. Furthermore, overexpression of BRD4 in HS578T cells, in which the baseline expression of mutant p53 is relatively low compared with other TNBC cells, also induced the expression of mutant p53 mRNA (*p* < 0.05, n = 3) (Figure 6C) and protein (Figure 6D).

### 2.4. Inhibition of BRD4 with JQ1 Results in Proliferative Defects in TNBC Cells Carrying Mutant p53 

**Figure 7:**
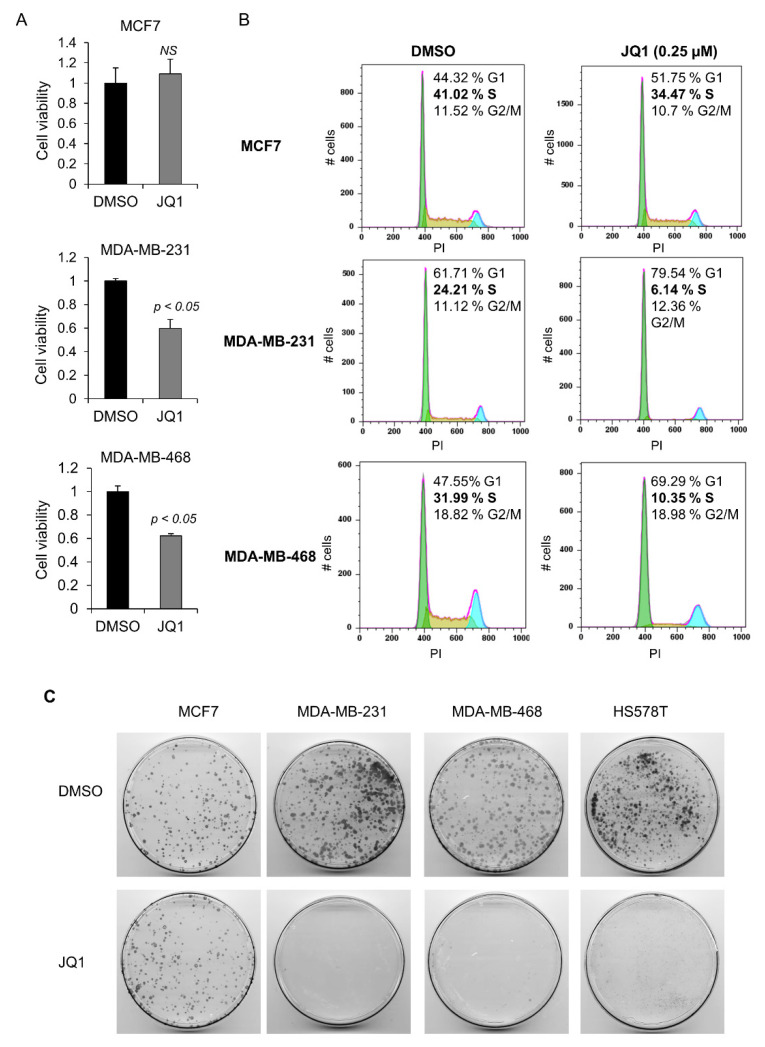
BET bromodomain inhibitor JQ1 induces G1 phase arrest and inhibits colony formation in TNBC cells with mutant p53. (**A**) MTS assay of MCF-7, MDA-MB-231, and MDA-MB-468 cells treated with 0.25 μM JQ1 for 72 h. All data are presented as the mean ± SEM; n = 3, *p* < 0.05 by Student’s *t*-test. NS, not significant. (**B**) MCF-7, MDA-MB-231, and MDA-MB-468 cells were treated with 0.25 μM JQ1 or DMSO for 24 h and were harvested for cell cycle analysis by PI staining. (**C**) For colony formation, MCF-7, MDA-MB-231, MDA-MB-468, and HS578T cells were cultured in medium with 0.25 μM JQ1 or DMSO for 15 days. The colonies were fixed and stained with 0.1% crystal violet.

To test whether JQ1 treatment inhibited the tumor cell growth, we performed a cell viability assay on TNBC cells and MCF-7 cells (control). Treatment with JQ1 (0.25 μM) for 72 h decreased the cell viability of TNBC cells (*p* < 0.05, n = 3), including MDA-MB-231 and MDA-MB-468, but had no obvious anti-proliferative effect in MCF-7 cells, as measured by MTS assay (Figure 7A). To further demonstrate the anti-proliferative effect of JQ1, the cell cycle profiles were analyzed by flow cytometry analysis. We found that the JQ1 treatment significantly decreased the percentage of cells in the S phase and increased the percentage of cells in the G1 phase in both MDA-MB-231 and MDA-MB-468 cells, but only slightly decreased the percentage of cells in the S phase in MCF-7 cells (Figure 7B). Furthermore, to determine the long-term effect of JQ1 on cell growth, we performed the colony formation assays of MCF-7 cells, as well as MDA-MB-231, MDA-MB-468, and HS578T cells treated with 0.25 μM JQ1. We found that the JQ1 treatment almost completely blocked the colony formation in the MDA-MB-231, MDA-MB-468, and HS578T cells with mutant p53s (Figure 7C). However, MCF-7 carrying wild-type p53 was less sensitive to JQ1 treatment (Figure 7C). Since p21 negatively regulates the S-phase entry and suppresses cell cycle progression [23], these results suggest that JQ1 treatment might inhibit the cell proliferation of TNBC cells through reducing mutant p53 and inducing p21 expression.

### 2.5. Knockdown of BRD4 Inhibits Tumor Growth of TNBC with Mutant p53 

**Figure 8:**
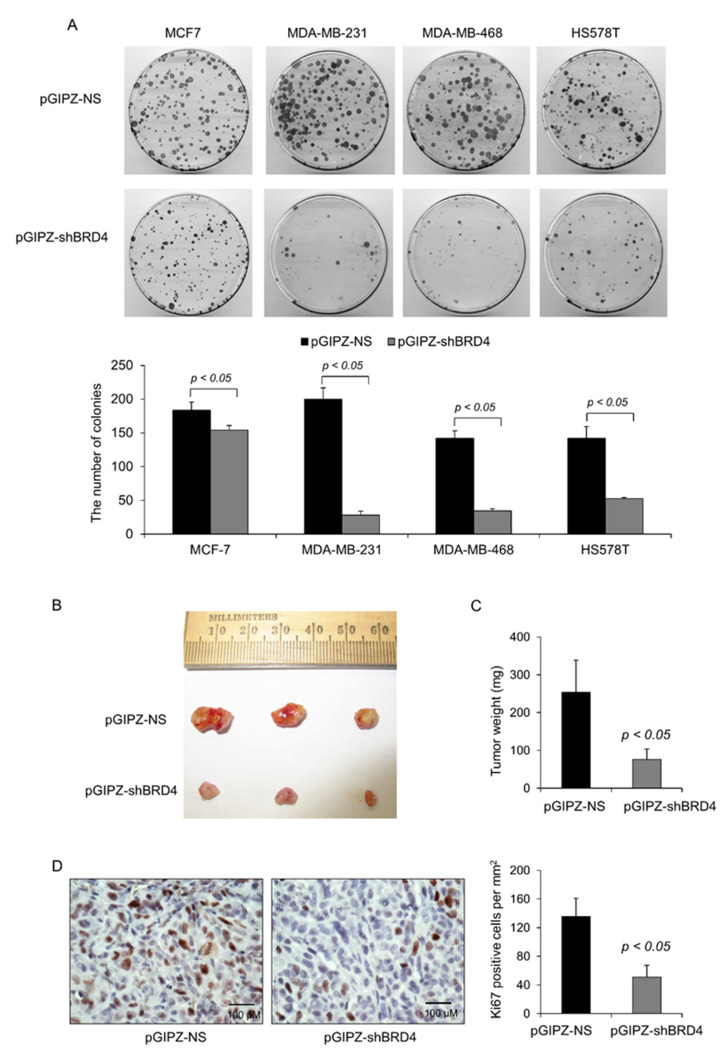
Knockdown of BRD4 inhibits tumor growth of TNBC cells in vitro and in vivo. (**A**) For colony formation, MCF-7, MDA-MB-231, MDA-MB-468, and HS578T cells were transduced with lentivirus expressing BRD4 shRNA or control shRNA and were cultured in a medium for 15 days. The colonies were fixed and stained with 0.1% crystal violet. Quantification of the number of colonies with a diameter >0.5 mm from three independent experiments (lower panel). All data are presented as the mean ± SEM; n = 3, *p* < 0.05 by Student’s *t*-test. (**B**,**C**) MDA-MB-231 cells transduced with lentivirus expressing BRD4 shRNA (n = 6) or control shRNA (n = 6) were inoculated into nude mice for xenograft tumor growth. Four weeks after inoculation, tumor xenografts were harvested and analyzed. All data are presented as the mean ± SEM; n = 6, *p* < 0.05 by Student’s *t*-test. (**D**) Knockdown of BRD4 decreased the proliferation of tumor xenografts, as analyzed by Ki67 staining. All data are presented as the mean ± SEM; n = 3, *p* < 0.05 by Student’s *t*-test.

To further support that BRD4 promotes TNBC cell growth, we generated the cells stably transduced with BRD4 shRNA or vehicle shRNA and performed the colony formation assays. In the TNBC cells carrying mutant p53s, knockdown of BRD4 resulted in an 86%, 76%, and 63% reduction in the number of colonies in MDA-MB-231, MDA-MB-468, and HS578T cells, respectively (*p* < 0.05, n = 3) (Figure 8A). However, knockdown of BRD4 in MCF-7 cells with wild-type p53 only led to a 15% reduction in the number of colonies (*p* < 0.05, n = 3) (Figure 8A). 

The MDA-MB-231 cell line was established from a pleural effusion of a patient with invasive ductal carcinoma—one of the most commonly studied cell lines—and is a highly aggressive and invasive in vivo breast cancer model [24]. The effect of BRD4 knockdown on suppressing colony formation in MDA-MB-231 cells was most obvious among the three TNBC cell lines (Figure 8A). Therefore, we next determined whether knockdown of BRD4 in MDA-MB-231 cells with shRNA inhibited tumor growth in vivo. MDA-MB-231 cells stably transduced with BRD4 shRNA or vehicle shRNA were inoculated into the mammary fat pad of nude mice. We found that tumor xenografts formed by MDA-MB-231 cells with BRD4 shRNA were much smaller than those formed by MDA-MB-231 cells with vehicle shRNA after 4 weeks of inoculation (*p* < 0.05, n = 6) (Figure 8B,C). The tumor cell proliferation was significantly decreased in tumors from MDA-MB-231 cells with BRD4 shRNA compared with those from MDA-MB-231 cells with vehicle shRNA, as examined by Ki67 staining (*p* < 0.05, n = 3) (Figure 8D). Together, these results suggest that knockdown of BRD4 inhibited TNBC cell proliferation and tumor growth through targeting the mutant p53 and the induction of p21.

## 3. Discussion

**Figure 9:**
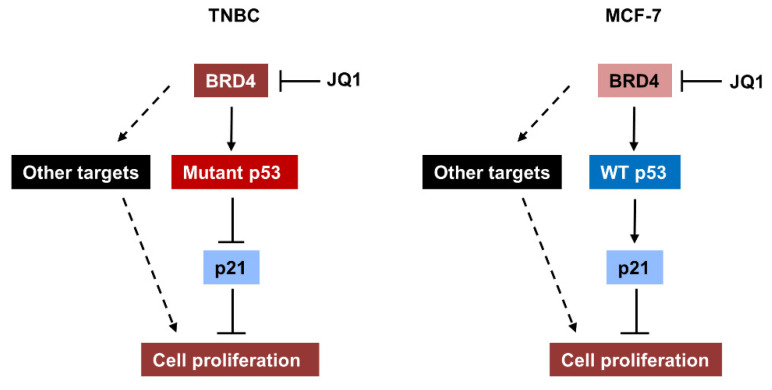
A schematic diagram depicting the BRD4/p53/p21 pathway in breast cancer. In TNBC cells, upregulation of BRD4 promotes the transcription of mutant p53, which suppresses the expression of p21, thereby increasing cell proliferation. In MCF-7 cells, BRD4 positively regulates the transcription of wild-type p53, which promotes the expression of p21. In addition, BRD4 might promote proliferation of breast cancer cells through modulating the expression of other targets.

In this study, we demonstrate for the first time that the BET protein BRD4 transcriptionally regulates the expression of mutant p53 in TNBC cells and that targeting BRD4 with JQ1 and shRNA decreased the p53 mRNA and protein expression in these cells through blocking the association of BRD4 with p53 promoter. These results suggest that, in addition to the increased stabilization, the ectopic transcription might result in the upregulation of mutant p53 in TNBC cells. Inhibition of BRD4 with JQ1 and knockdown of BRD4 increased the expression of p21 in TNBC cells but decreased the expression of p21 in MCF-7 cells. It has been reported that mutant p53 functions as dominant negative inhibitor over the wild-type p53 to inhibit the transcriptional activation of wild-type p53 target genes [4]. p21 is one of targets of wild-type p53 and an inhibitor of cyclin-dependent kinases, which is required for cell cycle progression during p53-mediated growth inhibition in many cell types [23]. Our results suggest that BRD4 mediated the expression of mutant p53 and has an opposite role to wild-type p53 in regulating the transcription of p21 in TNBC cells compared to MCF-7 cells (Figure 9). Furthermore, we found that knockdown of BRD4 in MDA-MB-231 cells with shRNA inhibited tumor growth in vivo. Additionally, the expression of BRD4 is upregulated, but the expression of p21 is downregulated in breast cancer samples with mutant p53 and the TNBC subclass from the TCGA datasets. 

BRD4 has been reported to associate with p53 in HEK293T cells and DNMT3A-mutated leukemia cells [25,26]. The interaction of BRD4 with p53 and the access of BRD4 to the acetylated chromatin is regulated by casein kinase II (CK2) mediated the phosphorylation of BRD4 [26]. Phospho-BRD4 co-occupied with p53 at the promoter of p21 that contains a distal p53 binding site [26]. Knockdown of BRD4 with shRNA decreased the mRNA levels of p21 in HEK293T and HCT116 cells—a human colon cancer cell line—in a p53-dependent manner [26]. In this study, we found that, in addition to directly interacting with p53, BRD4 also transcriptionally regulates the expression of wild-type and mutant p53 in breast cancer cells. Targeting BRD4 also resulted in a decrease in p21 mRNA and protein in MCF-7 cells carrying wild-type p53, suggesting that wild-type p53 regulates the transcription of p21 positively in MCF-7 cells. However, inhibition or knockdown of BRD4 increased the expression of p21 in TNBC cells. It has been reported that the p53 mutants suppress a variety of p53 target genes through inducing hypo-acetylation of these promoters [22]. Thus, the downregulation of mutant p53 by knockdown of BRD4 and JQ1 treatment might release its suppressive effect on the p21 promoter. However, a mechanism other than mutant p53 may also be involved in BRD4-mediated p21 expression in TNBC cells, which needs to be further investigated. 

Mutant p53, which is usually upregulated in cancer cells, possesses pro-oncogenic potential through gain of functions such as enhanced invasion, motility, transcriptional repression, and activation. Thus, targeting mutant p53 and mutant p53-mediated pathways has become a promising strategy and are being explored in the development of novel anti-cancer agents [4,27]. One strategy to targeting mutant p53 is to promote the degradation of mutant p53. The interaction of mutant p53 with the Hsp70 and Hsp90 chaperone complex stabilizes the mutant p53, which is dependent on the association with HDAC6 [28,29]. Inhibition of HDAC6 with HDAC inhibitor SAHA results in the disassociation of mutant p53 from the chaperone complex, leading to its degradation [28,29]. In addition to inducing the degradation of mutant p53, another strategy is to restore wild-type p53 function and to increase the level of p53 with a wild-type conformation and activity to induce apoptosis and senescence through binding to multiple mutant p53 proteins [30,31,32]. Furthermore, the downstream pathways activated by mutant p53 have also been targeted for therapeutic intervention. Inhibition of the cholesterol synthesis pathway, which is activated by mutant p53, restored the morphology and decreased the survival of mammary cancer cells with mutant p53 [33]. We demonstrated here that the transcription of mutant p53 was increased in TNBCs via BRD4 and targeting BRD4 with JQ1 decreased the transcription of mutant p53 and the growth of TNBC cells. The limitation of our study is that the TNBC patient samples are not included to validate these findings. However, the bioinformatic analysis of TNBC data from the TCGA database suggests that BRD4 is upregulated in breast cancer samples with mutant p53 and TNBC subclasses (Figure 1D–E). In addition to BRD4, the expression or stabilization of mutant p53 could also be regulated by other epigenetic regulators, such as HDAC6, HDAC8, SIRT1, and EZH2, through different mechanisms [7,8,9,28], suggesting that targeting these epigenetic regulators might also be a possible approach to target mutant p53 in TNBC. It has been reported that HDAC inhibitors in combination with other anticancer reagents have shown promising therapeutic outcomes against TNBC [34]. BET and HDAC inhibitors also synergize to decrease the progression of Myc-induced murine lymphoma through inducing similar genes and biological effects [35]. We believe that a synergistic effect of BET and HDAC inhibitors by targeting mutant p53 exists in TNBCs.

Our results that knockout of BRD4 significantly blocked colony formation in the TNBC cells lines MDA-MB-231, MDA-MB-468, and HS578T carrying mutant p53, while it only slightly decreased the colony formation of MCF-7 cells that carry wild-type p53, suggesting that the sensitivity of the breast cancer cells’ response to the BET inhibitor might be dependent on the status of p53. Thus, an effective personalized cancer therapy by BETi requires not only a thorough understanding of the mechanism of action of the BETs but also a thoughtful consideration of the genetic and epigenetic alterations for each individual cancer, such as the status of *TP53*. On the other hand, knockdown of BRD4 with shRNA or inhibition of BRD4 with JQ1 decreased the expression of p53 and p21—two important proteins of cell cycle arrest—in MCF-7 cells. JQ1 treatment still inhibited the colony formation of MCF-7 cells despite it decreasing the level of wild-type p53 and p21. This observation demonstrated that BRD4 regulated the tumor growth in breast cancer cells with wild-type p53 through mechanisms other than p53/p21 signaling, such as enhancer activation and gene transcription of c-Myc and estrogen receptor [36,37], which needs to be further investigated. 

The treatment of TNBC is limited due to its poor prognosis and aggressiveness, as well as due to the lack of targeted therapies. Therefore, it is essential to identify new therapeutic approaches by understanding the mechanisms, molecules, and signaling pathways that participate in the epigenetic modulation of genes expressed in carcinogenesis in TNBC. Our study showed that JQ1 inhibited the proliferation of three TNBC cell lines in vitro, and knockdown of BRD4 with shRNA in TNBC cell line MDA-MB-231 inhibited tumor growth in xenograft models. Several studies have reported that JQ1 treatment inhibited growth of TNBC cells with different mechanisms, including induction of apoptosis or senescence, inhibition of cell proliferation, downregulation of transcription factors (FOXM1, E2F8, LIN9, MYBL2), and disruption of hypoxia-associated factors and decreased angiogenesis [38,39,40,41]. Our findings elucidated a novel mechanism in that targeting of BRD4 inhibited tumorigenesis through downregulation of mutant p53 in TNBC cells, suggesting that BETi may have a higher efficacy in TNBC patients with mutant p53. Our findings further suggest that targeting BET proteins with inhibitors together with currently used chemotherapy and/or immunotherapy may be potential therapies for TNBC treatment. Several ongoing clinical trials are designed to test the combination of BET inhibitor ZEN003694 with other therapies in triple-negative breast cancer, including ZEN003694 and MAPK inhibitor Binimetinib (NCT05111561), ZEN003694 and PARP inhibitor Talazoparib (NCT03901469), and ZEN003694 and PD-1 inhibitor Pembrolizumab, in addition to the standard chemotherapy (NCT05422794).

In sum, our study details a mechanism of transcriptional regulation of p53 and p21 by BRD4 in TNBC and breast cancer cells. Due to the high frequency of p53 mutation and the requirement of BRD4 for mutant p53 transcription, our results suggest that targeting BRD4 holds promise as a potential therapeutic modality for TNBC carrying a mutant p53. 

## 4. Materials and Methods

### 4.1. Cell Culture and Reagents 

Human breast cancer cell lines MCF-7, MDA-MB-231, MDA-MB-468, HS578T, and Human Embryonic Kidney 293 cells (HEK293) were obtained from American Type Culture Collection (ATCC). All of the cells were maintained according to the recommended protocol. JQ1 was purchased from MedChem Express and dissolved in DMSO (Sigma). pCMV-Flag-BRD4 plasmid were purchased from Addgene. 

MCF-7, MDA-MB-231, MDA-MB-468, and HS578T cells treated with 1 μM JQ1 or 0.1% (*v*/*v*) DMSO for 24 h were harvested for RNA extraction. MCF-7, MDA-MB-231, MDA-MB-468, and HS578T cells treated with different concentrations of JQ1 (0 μM, 0.125 μM, 0.25 μM, 0.5 μM, 1 μM, and 2 μM) for 72 h were harvested for protein extraction and Western blot analysis. MDA-MB-231, MDA-MB-468, and HS578T cells treated with 1 μM JQ1 for different time periods (0 h, 4 h, 8 h, 12 h, and 24 h) were harvested for protein extraction and Western blot analysis.

### 4.2. The Cancer Genome Atlas (TCGA) Data Mining 

UALCAN (http://ualcan.path.uab.edu, accessed on 7 November 2022) is a comprehensive interactive web resource for analyzing cancer OMICS data [18,19]. The gene expression of BRD4 and p21 in breast cancer based on p53 mutation status and breast cancer subclasses was analyzed by UALCAN.

### 4.3. BRD4 Knockdown by Lentivirus Carrying BRD4 shRNA 

HEK293T cells were transfected either with lentiviral plasmid pGIPZ-shBRD4 (Open Biosystems) or with a control empty vector pGIPZ-NS, plus psPAX2 packaging plasmid and pMD2.G envelope plasmid using calcium phosphate. Sixteen hours after transfection, the medium containing the transfection reagent was removed and replaced with fresh complete DMEM plus 10% FBS and penicillin/streptomycin. The lentiviral particles were harvested from the supernatant of transfected HEK293T cells after another 48 h. MCF-7, MDA-MB-231, MDA-MB-468, and HS578T cells were infected with appropriate amounts of lentiviral particles together with 5 μg/mL polybrene (Sigma-Aldrich, St. Louis, MI, USA), and then selected by 10 μg/mL puromycin until all remaining cells were GFP positive, as detected by microscopy. The cells were harvested after lentiviral particle infection for 5 days and analyzed by RT-PCR and Western blot to examine their efficiency in BRD4 knockdown.

### 4.4. Overexpression of BRD4 

Plasmids of pCMV-Flag-BRD4 and the empty vector control were transfected into MCF7 cells or HS578T cells. After transfection for 48 h, cells were harvested and analyzed by qRT-PCR and Western blot analysis.

### 4.5. Western Blot Analysis 

We performed Western blotting on whole-cell lysates as previous described [42]. Briefly, cell pellets were collected and re-suspended in lysis buffer (20 mM Tris-HCl, pH 7.4, 150 mM NaCl, 10% glycerol, 1% Triton X-100, 1 mM Na_3_VO_4_, 25 mM β-glycerol-phosphate, 0.1 mM PMSF, Roche complete protease inhibitor set, and Sigma phosphatase inhibitor set). The re-suspended cell pellet was vortexed for 20 s and then incubated on ice for 30 min and centrifuged at 20,000× *g* for 30 min. The protein in the cell lysates were subjected to Western blot analysis. The antibodies used for Western analysis included anti-p53, anti-p21 antibodies (Santa Cruz, Dallas, TX, USA, 1:500 dilution), anti-BRD4 (Bethyl, Waltham, USA, 1:1000 dilution), and anti-actin antibody (Sigma, St. Louis, MI, USA, 1:5000 dilution). Donkey-anti-rabbit IgG-horseradish peroxidase and Goat anti-mouse IgG-horseradish peroxidase (Santa Cruz, 1:8000 dilution) were used as secondary antibodies. 

### 4.6. Quantitative Reverse-Transcription Polymerase Chain Reaction (qRT-PCR)

Total RNA was extracted using the RNeasy plus mini kit (Qiagen). Total RNA (1 μg) was used for RT reactions in a 20-μL reaction to synthesize cDNA using Iscript cDNA Synthesis Kit (BioRad, Hercules, CA, USA). RNA expression profiles were analyzed by real-time PCR using iTaq SYBER Green Supermix (BioRad) in a CFT connect real-time PCR detection system (BioRad). The complete reactions were subjected to the following program of thermal cycling: 40 cycles of 10 s at 95 °C and 30 s at 60 °C. A melting curve was run after the PCR cycles, followed by a cooling step. Each sample was run in triplicate in each experiment. The relative gene expression levels of target genes were analyzed by 2^−ΔΔ*C*^_T_ method by using two reference genes, GAPDH and CCSER2 [43]. The primers used were: p53-F 5′-ATTCTGCCCTCACAGCTCTGGCT-3′; p53-R; 5′-CCGGAGGAAGCAAAGGAAATGG-3′; p21-F 5′- TGTCACTGTCTTGTACCCTTG-3′; p21-R 5′- GGCGTTTGGAGTGGTAGAA-3′; BRD4-F5′-AGGCAAAAGGAAGAGGACG-3′; BRD4-R5′- CGATGCTTGAGTTGTGTTTGG-3′; GAPDH-F 5′-GGATTTGGTCGTATTGGG-3′; GAPDH-R 5′-GGAAGATGGTGATGGGATT-3′; CCSER2-F 5′-GACAGGAGCATTACCACCTCAG-3′; and CCSER2-R 5′-CTTCTGAGCCTGGAAAAAGGGC-3′.

### 4.7. Chromatin Immunoprecipitation (CHIP)-qPCR Assay

The ChIP assay was performed according to the protocol [44]. Chromatin DNA was subjected to immunoprecipitation (IP) with anti-BRD4, anti-RNA polymerase II, anti-Histone 3 antibodies, or normal rabbit IgG and then washed, after which the DNA-protein cross-links were reversed. The recovered DNA was analyzed by qPCR by using the following PCR primers: p53 promoter-F: 5′-CAGGTCGGCGAGAATCCTG-3′, p53 promoter-R: 5′-TGGCTCTAGACTTTTGAGAAGC-3′.

### 4.8. MTS Assay

MCF-7, MDA-MB-231, and MDA-MB468 cells (5000 cells in 100 μL culture medium) were dispensed in each well of a 96-well assay plate. The cells after incubation overnight were treated with 0.25 μM JQ1 or 0.1% (*v*/*v*) DMSO for 72 h (n = 3 in in each group). Cell viability was measured by using CellTiter 96 AQ_ueous_ One Solution Cell proliferation Assay (MTS) kit (Promega), according to the manufacturer’s instructions. Briefly, 20 μL of CellTiter 96 AQ_ueous_ One Solution Regent were pipetted into each well of the 96-well assay plate. After incubation of the plate at 37 °C for 4 h, the absorbance at 490 nm were recorded by a 96-well plate reader. 

### 4.9. Cell Cycle Analysis

A total of 150,000 cells were seeded in six-well plates and allowed to recover for 16–24 h; cells were then starved with serum-free medium for 24 h. Then cells were left untreated or treated with 0.25 μM JQ1 for 24 h. The cells were harvested and fixed in 70% cold ethanol at −20 °C overnight. For PI staining, after three times wash with PBS, the cells were suspended in 0.5 mL of PI buffer (containing RNase and Triton X-100) for 10–15 min at room temperature. The cells were analyzed with a flow cytometer, and FACS data were analyzed with FlowJo software.

### 4.10. Colony Formation Assay

MCF-7, MDA-MB-231, MDA-MB-468, and HS578T cells were seeded at a concentration of 1000 cells per dish with the culture medium with 0.25 μM JQ1 or DMSO. After incubation for 15 days, the cells were washed and fixed in methanol for 15 min at −20 °C and stained with 0.1% crystal violet. The colonies were then counted, photographed, and quantified. MCF-7, MDA-MB-231, MDA-MB-468, and HS578T with BRD4 shRNA or vehicle shRNA were used to perform the colony formation assay as described above.

### 4.11. Mice Xenograft Studies

All animal studies were done in accordance with a protocol approved by the Institutional Animal Care and Use Committee (IACUC) of the University of Kansas Medical Center. Animals were maintained and treated under pathogen-free conditions. Female NCI-nu/nu nude (Charles River) of 4–6 weeks old were used in xenograft studies orthotopic mammary fat pad tumor assays. MDA-MB231 cells with BRD4 shRNA or vehicle shRNA were collected in PBS and inoculated into the right mammary fat pad (5 × 10^7^/mL, 0.1 mL per mouse, n = 6 in each group). Four weeks after the inoculation, mice were euthanized, and tumors were removed and weighed.

### 4.12. Immunohistochemistry Staining

Tumor xenograft tissues were fixed with 4% paraformaldehyde (pH 7.4) and embedded in paraffin. For Ki67 staining, a monoclonal mouse anti-Ki67 antibody (Abcam; 1:1000 dilution), a biotinylated secondary antibody (Sigma-Aldrich; 1:100 dilution), and DAB substrate system were used. Then, sections were counter stained by hematoxylin. Images were analyzed with a NIKON ECLIPSE 80i microscope. The number of Ki67-positive cells per mm^2^ tumor tissue was counted. 

### 4.13. Statistics

Data from the TCGA dataset are presented in box–whisker plots, which present the interquartile ranges (IQRs), including the minimum, 1st quartile, median, 3rd quartile, and maximum values. A Welch’s *t*-test was used to determine the significance of the differences. All other data are presented as the mean ± SEM. An unpaired 2-tailed Student’s t-test was used to determine the significance of the differences. A *p* value less than 0.05 was considered significant.
